# Value Judgments in Mathematics: G. H. Hardy and the (Non-)seriousness of Mathematical Theorems

**DOI:** 10.1007/s10516-023-09705-y

**Published:** 2024-02-14

**Authors:** Simon Weisgerber

**Affiliations:** Department of Philosophy, University of Vienna, Universitätsstraße 7, 1010 Vienna, Austria

**Keywords:** G. H. Hardy, A mathematician’s apology, Values in mathematics, Serious mathematics, Mathematical interest, Reverse multiples

## Abstract

One of the general criteria G. H. Hardy identifies and discusses in his famous essay *A Mathematician’s Apology*, Cambridge University Press, Cambridge, 1940) by which a mathematician’s patterns must be judged is *seriousness*. This article focuses on one of Hardy’s examples of a non-serious theorem, namely that 8712 and 9801 are the only numbers below 10000 which are integral multiples of their reversals, in the sense that $8712=4\cdot 2178$, and $9801=9\cdot 1089$. In the context of a discussion of *generality*, which he considers an essential quality of seriousness, he explains that there is nothing in this example which “appeals much to a mathematician” and that it is “not capable of any significant generalization.” Interestingly, since the publication of the *Apology*, more than a dozen papers—including one by the renowned mathematician Neil Sloane—have been published that discuss generalizations of Hardy’s example. By identifying the most important aspect of Hardy’s notion of generality, it is argued that, contrary to the views of several researchers, Hardy’s claim regarding the non-capability of any significant generalization is still tenable. Furthermore, this case study is presented and discussed as an example of the multifaceted nature of *mathematical interest*.

## Introduction

In his famous essay *A Mathematician’s Apology* from 1940, the English mathematician Godfrey Harold Hardy (1877–1947) provides a justification for a serious study of mathematics and, with it, for the life of a professional mathematician. Hardy argues that mathematics must be justified as a *creative art*. As he famously puts it: “Beauty is the first test: there is no permanent place in the world for ugly mathematics” (1940/2012, p. 85). The general criteria Hardy identifies and discusses in his essay by which a mathematician’s patterns must be judged are *beauty* and *seriousness*.

References to Hardy’s thoughts presented in his essay readily appear in the works of philosophers and researchers in mathematics education when investigating, for instance, mathematical problem choice (Ashton 2018), whether mathematics can be treated as an art (Tymoczko 1993), values in mathematics ((Ernest 1998), (Weisgerber 2023)) and aesthetic considerations in mathematical inquiry more generally (Sinclair 2004, 2006, 2011) or mathematical beauty ((Dutilh Novaes 2019), (Sa et al. 2023)) and depth (Urquhart 2015) in particular.

In this article, I focus on Hardy’s notion of seriousness and in particular on a specific example proposed by him (example (*a*) below) as an example of a *non-serious* mathematical theorem. Roughly speaking, a serious theorem is a theorem which connects *significant* mathematical ideas, while a significant idea is an idea which “can be connected, in a natural and illuminating way, with a large complex of other mathematical ideas” (Hardy 1940/2012, p. 89).

One quality that Hardy says is essential to the significance of a mathematical idea or the seriousness of a theorem is *generality*. While discussing this quality, he presents the following two examples that (allegedly) lack this quality “conspicuously” (in ways we will discuss in more detail later) and therefore cannot be serious (ibid., pp. 104f.):

(*a*): 8712 and 9801 are the only four-figure numbers which are integral multiples of their ‘reversals’:
  $$\begin{aligned} 8712=4\cdot 2178,\, 9801=9\cdot 1089, \end{aligned}$$
  and there are no other numbers below 10,000 which have this property.
(*b*): There are just four numbers (after 1) which are the sums of the cubes of their digits, viz.
  $$\begin{aligned} \begin{aligned}&153=1^3+5^3+3^3,\, 370=3^3+7^3+0^3, \\&371=3^3+7^3+1^3,\, 407=4^3+0^3+7^3. \end{aligned} \end{aligned}$$

After having introduced these examples, Hardy states that (hereinafter referred to as $(\mathcal {D})$, where “$\mathcal {D}$” is meant to remind of “diagnosis”)

$$(\mathcal {D})$$
These are odd facts, very suitable for puzzle columns and likely to amuse amateurs, but there is nothing in them which appeals much to a mathematician. The proofs are neither difficult nor interesting—merely a little tiresome. The theorems are not serious; and it is plain that one reason (though perhaps not the most important) is the extreme speciality of both the enunciations and the proofs, which are not capable of any significant generalization. (ibid., p. 105)

Interestingly, since the publication of Hardy’s *Apology*, more than a dozen papers—including one by the renowned mathematician Neil Sloane—have been published on the phenomenon of *reverse multiples*, i.e., on numbers with the special property described in example (*a*), that discuss nontrivial (in terms of difficulty) generalizations.

My main goal in this article is to clarify what Hardy meant by his diagnosis and to examine it for accuracy, especially in light of the research that has been done since his claims were made. In doing so, I will, among other things, identify and argue for what I believe to be the most important aspect of Hardy’s notion of generality. Taking this result into account, I will further argue that his upshot that example (*a*) is “not capable of any significant generalization” is still tenable, which contradicts several authors dealing with the phenomenon of reverse multiples—again including Sloane—who explicitly refer to (part of) $(\mathcal {D})$. Finally, I will present and discuss this phenomenon as an example of the multifaceted nature of *mathematical interest*.

At this point, a few words should be said about Hardy’s *Apology* and its cultural and historical context. Hardy wrote on it as a professor at Cambridge during the early months of World War II. One of the concluding sections of the *Apology*, namely § 28, is based on Hardy’s article “Mathematics in War-Time” which was published in January 1940, where he states with respect to the functions of mathematics in war that they fill him “with intellectual contempt and moral disgust” (1940, p. 5). A major theme in his justification of a serious study of mathematics presented in his *Apology* is the (alleged) *harmlessness*—in terms of the effects on war—of (real) mathematics. Hardy’s essay can thus not only be regarded as a philosophical piece, but in some ways as a political one as well.

In general, Hardy was not unique in his time in justifying mathematics in his *Apology*—or earlier in his career (at least indirectly), e.g., in his inaugural lecture he gave in Oxford (Hardy 1920)—on aesthetic grounds rather than with reference to possible applications. There has already been a tradition among British pure mathematicians, at least since the last decades of the 19th century, to justify their mathematics by appeal to its aesthetics (cf. (Heard 2019, Chapter 7)).

The structure of this paper is as follows. In Sect. 2, some of the main research results on the phenomenon of reverse multiples are presented. After having introduced, in Sect. 3, Hardy’s main ideas on seriousness relevant to the present study, a closer look is taken at his notion of generality in Sect. 4. First, Hardy’s proposed ways in which (*a*) lacks generality are discussed (4.1). Then, the most important aspect of Hardy’s notion of generality is identified (4.2). Finally, in Sect. 5, diagnosis $(\mathcal {D})$ is discussed by, among other things, evaluating researchers’ interpretations (5.1) and by examining mathematical interests in reverse multiples (5.2).

## The General Phenomenon of Reverse Multiples

In this section, I present some of the main research that has been carried out on the general phenomenon of reverse multiples. In order to provide some self-containment, I will cover Ludington Young’s (1992a) contribution and, to a certain degree that of Sloane (2014), in some detail.

Sloane listed in (2014) all the work on the general phenomenon of reverse multiples of which he was aware at the time. His earliest references are two brief responses published in volume XV of *L’Intermédiaire des mathématiciens* in 1908 (Laisant et al. 1908, pp. 132f., 278f.) to a problem that has been posed in volume VI of the same journal published in 1899. There, J. Jonesco asks, among other things, “[w]hat are then the numbers *n* smaller than the base of the system, such that there are products *n*N which are the reversals of N?”^1^ (Laisant and Lemoine 1899, p. 200, problem 1622). So, Jonesco was not just interested in the base-10 case, as in Hardy’s example (*a*). One of the respondent gave two examples of this general case, namely 1015 in base 8, since $5\cdot 1015=5101$, and 18 in base 13, since $5\cdot 18=81$.

Alan Sutcliffe’s (1966) “Integers that are multiplied when their digits are reversed” published in *Mathematics Magazine*, appears to be the first paper after the publication of Hardy’s *Apology* that discusses generalizations of reverse multiples (see also (Sloane 2014, p. 99)). In this work, Sutcliffe is interested in the general phenomenon of base-*g* reverse multiples, i.e., he is interested in the solution of

1 $$\begin{aligned}{} & {} k(a_{n-1}g^{n-1}+a_{n-2}g^{n-2}\nonumber \\{} & {} \quad +\cdots +a_1g+a_0)=a_0g^{n-1}+a_1g^{n-2}+\cdots +a_{n-2}g+a_{n-1} \end{aligned}$$

where $k,a_0,\dots ,a_{n-1}$ are all less than *g* and $k>1$, $a_0>0$ and $a_{n-1}>0$.^2^ He is mainly concerned with a characterization of 2- and 3-digit numbers. For 2-digit numbers he proves, for instance, that there is a solution of Equation (1) in base $g>3$ if and only if $g+1$ is non-prime. He notes that things are problematic for numbers with more digits since “for each additional digit there is one new equation but two new variables” (1966, p. 287). He also conjectured that if there exists a 3-digit solution for the base *g*, then there also exists a 2-digit solution for *g*, but explains that “no general proof has been found” (ibid., p. 285). T. J. Kaczynski (1968)—who is, as Lara Pudwell (2007, p. 129) puts it, “[b]etter known for other work”—proved that this conjecture is correct. Based on Sutcliffe and Kaczynski’s results, Pudwell (2007) asks whether there is any value of *g* for which there is a 5-digit solution but no 4-digit solution which she answers in the negative.

**Fig. 1:**
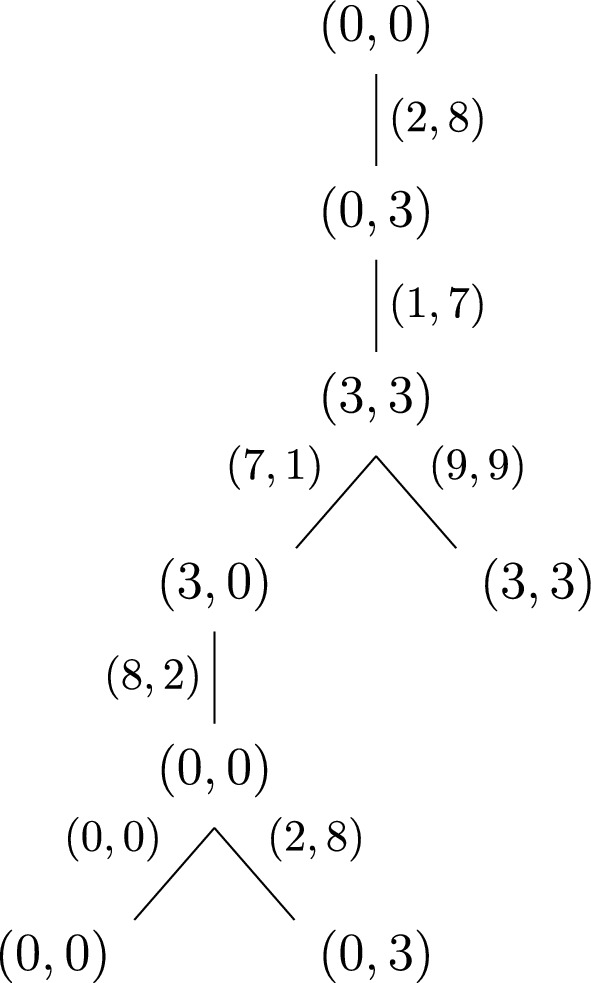
Pruned rooted tree for $g=10$ and $k=4$

In Anne Ludington Young’s (1992a; b) two articles on *k*-reverse multiples, which appeared in the same issue of *The Fibonacci Quarterly*, she focuses on the task of determining *all* *k*-reverse multiples in base *g*, for given *k* and *g*, once those with a small number of digits are known. To this end, she introduces a graphical notation in the form of specific rooted trees (see as an example Fig. 1). By comparing corresponding digits of Equation (1), she gets the following set of equations: 
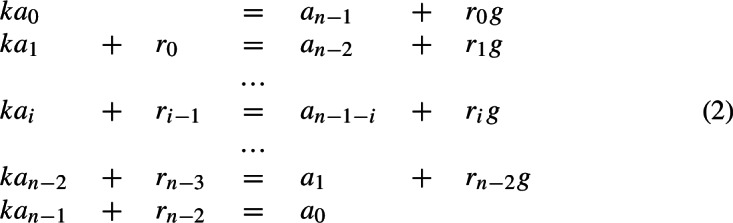


where $0\le r_i<g$ for $i=0,\dots ,n-2$. The $r_i$’s are called the “carry numbers” or simply “carries.” For instance, if we think of Hardy’s first mentioned reverse multiple, namely 2178, we have $(r_2,r_1,r_0)=(0,3,3)$; for example, the first equation of (2) is $4\cdot 8=2+{\textbf {3}}\cdot 10.$ Now, Ludington Young considers two equations of (2) at a time. She sets $r_{-1}=r_{n-1}=0$ for convenience. At the $(i+1)$-st step for $i=0,1,\dots$, one has 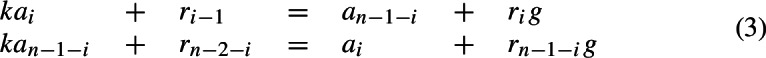


where $r_{i-1}$ and $r_{n-1-i}$ are known from the previous step. If the non-negative numbers in (3) also satisfy

4 $$\begin{aligned} a_0,a_{n-1}>0,\,a_i<g\,\textrm{for}\, i=0,1,\dots ,n-1,\,r_0>0\,\textrm{and}\,r_i<k\,\textrm{for}\,i=0,\dots ,n-2,\nonumber \\ \end{aligned}$$

then Ludington Young writes 
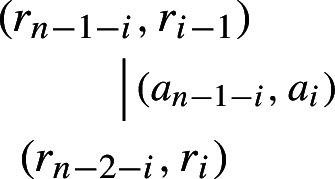


and conversely. That is to say that the existence of non-negative solutions to (3) and (4) is equivalent to the existence of a tree of the form 
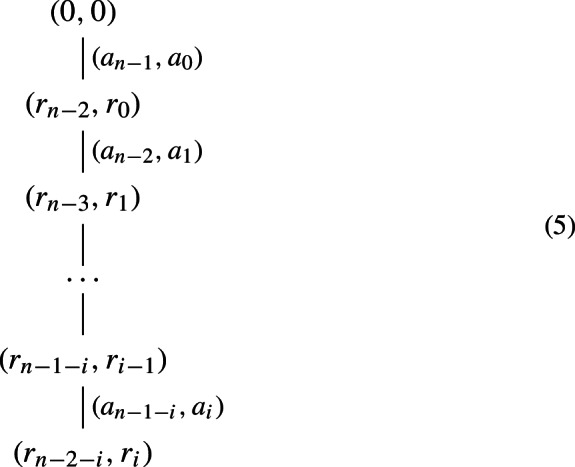
 As an example, Ludington Young (1992a, p. 128, Example 1) considers the case for $g=10$ and $k=4$ for which she gets the graph or rooted tree shown in Fig. 1 Note that although these trees are in essence infinite, the tree in Fig. 1 is pruned after the nodes from which no new information would emerge.

Now, how do we use these trees to determine *k*-reverse multiples? Ludington Young answers this with the help of two theorems:

Theorem 1 (Ludington Young 1992a, Theorem 1, p. 129): For a given *g* and *k*, suppose a tree of the form (5) exists. Then there is an $n=2i+2$-digit number *x* satisfying (1) if and only if $r_{n-2-i}=r_i$. In this case, *x* is given by

$$\begin{aligned} x=a_{n-1}a_{n-2}\dots a_{n-1-i}a_i\dots a_1a_0. \end{aligned}$$

The theorem says that if there is a tree of the form (5) with a path of length *n*/2 for an even number *n* from the root to a node of the form (*u*, *u*), then there exists an *n*-digit reverse multiple. Given a tree with such a node, we can then simply read off the corresponding reverse multiple by first listing all the left entries of the labels of the edges while following the path until we reach the node of the form (*u*, *u*), and then, while going back to the root, listing all the right entries of these labels.

As an example, consider Fig. 1. The path from the starting node (0, 0) to the first appearance of the node (3, 3) gives us the number 2178, which is one of the four-digit reverse multiples from Hardy’s example (*a*). We could also pick the path from the root to the second appearance of the node (3, 3). In this case, we would get the reverse multiple 219978. Note that the tree in Fig. 1 is pruned; there are infinitely many more 4-reverse multiples in base 10.

Theorem 2 (Ludington Young 1992a, Theorem 3, p. 130): For a given *g* and *k*, suppose a tree of the form (5) exists. Then there is an $n=2i+3$-digit number *x* satisfying (1) if and only if the graph contains 
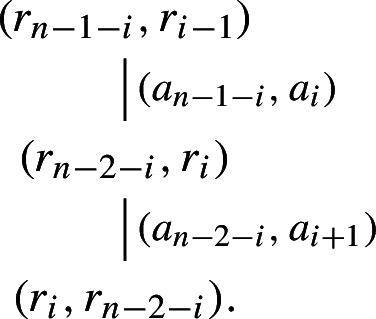


Further, when this occurs, $a_{n-2-i}=a_{i+1}=M=(r_{n-2-i}g-r_i)/(k-1)$. The desired *n*-digit number *x* is given by

$$\begin{aligned} x=a_{n-1}a_{n-2}\dots a_{n-1-i}Ma_i\dots a_1a_0. \end{aligned}$$

In Fig. 1, there is an edge from (3, 3) to (3, 3) which ensures the existence of reverse multiples with an odd number of digits according to the previous theorem, such as 21978. In this case, $a_{n-2-i}=a_{i+1}=M=9$.

In her follow-up article (1992b), Ludington Young examines these trees in more detail. Her main results are essentially of the type that if one or more labelled edges are known along with their nodes, then other parts of the tree are also known in principle.

**Fig. 2:**
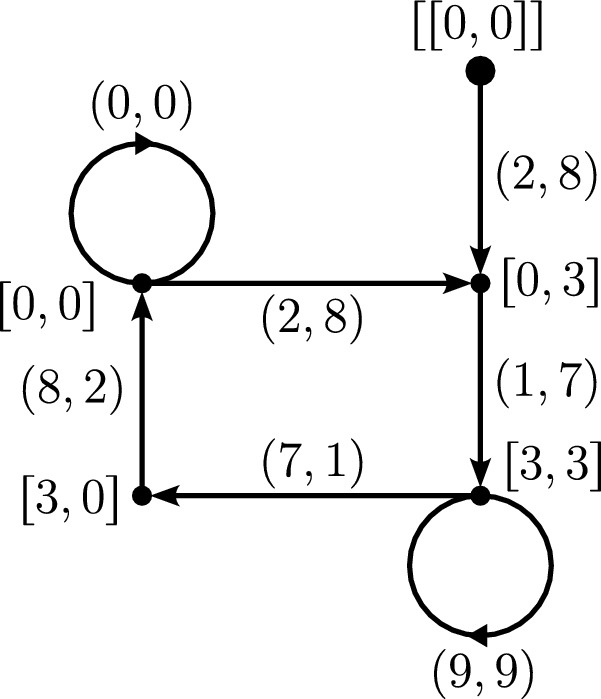
The (10, 4) Young graph

The mathematician Neil Sloane, who is best known for his role as the founder and maintainer of the *On-Line Encyclopedia of Integer Sequences* (OEIS) (The OEIS Foundation Inc. 2023), extends Ludington Young’s work in (Sloane 2014), which also appeared in *The Fibonacci Quarterly*. He introduces, inter alia, modified versions of Ludington Young’s trees, which are finite, directed graphs and which he calls *Young graphs*, and determines the possible values of *k* for bases $2\le g\le 100$. Further, he shows how to use the *transfer-matrix method* from combinatorics (by applying it to the Young graphs) in order to enumerate the (*g*, *k*)-reverse multiples with a given number of base-*g* digits. The Young graphs result from Ludington Young’s pruned rooted trees by essentially identifying equal nodes, except for the starting node and other occurrences of (0, 0). For instance, the pruned tree of Fig. 1 gives the Young graph shown in Fig. 2 (2014, p. 104, Figure 4) (Note that Sloane uses square brackets for the nodes and double brackets for the starting node.):

Sloane (2014) shows, among other things, how to apply the transfer-matrix method to these Young graphs in order to determine their *generating functions*, i.e., formal power series of the form

$$\begin{aligned} \mathcal {C}(x)=\sum _{t\ge 0}c_tx^t, \end{aligned}$$

where the $c_t$’s denote the numbers of (*g*, *k*)-reverse multiples with *t* digits. One of his concrete examples is the generating function for the number of *all* base-10 reverse multiples, regardless of the multiplier. He gets (2014, p. 109)

6 $$\begin{aligned}{} & {} \mathcal {C}(x)=2\sum _{t=4}^\infty F_{\lfloor \frac{t}{2}\rfloor -1}x^t=2x^4+2x^5+2x^6\nonumber \\{} & {} \quad +2x^7+4x^8+4x^9+6x^{10}+6x^{11}+10x^{12}+10x^{13}+\cdots , \end{aligned}$$

where $F_{\lfloor \frac{t}{2}\rfloor -1}$ is the $(\lfloor \frac{t}{2}\rfloor -1)$-st Fibonacci number. So, in the base-10 case, the reverse multiples are basically enumerated by the Fibonacci numbers.^3^ Note that the first 2, i.e., the coefficient of $x^4$, corresponds to the two reverse multiples in Hardy’s example (*a*).

In general, Sloane (2014) identifies and characterizes in more detail three particular families of Young graphs. For example, one such family is the isomorphism class of the (10, 4) Young graph, which also contains the (10, 9) Young graph. Note that two Young graphs are isomorphic if and only if their underlying directed graphs are the same and some particular nodes of both graphs, each playing the same role in identifying the reverse multiples, correspond to each other.

Furthermore, he presents several computer-generated results for bases $g\le 20$. For example, all (*g*, *k*) values for which reverse multiples exist in this case are listed along with a specification of the respective Young graphs (see (ibid., p. 115, Table 1)).

Sloane also raises some open questions and makes several conjectures, some of which have been addressed by Lev Kendrick (2015). As Kendrick himself explains in the abstract of his article:

We prove Sloane’s isomorphism conjectures for 1089 graphs [i.e., graphs that are isomorphic to the (10, 4) and (10, 9) Young graphs; S.W.] and complete graphs, namely that the Young graph for *g* and *k* is a 1089 graph if and only if $k+1$ | *g* and is a complete Young graph on *m* nodes if and only if $\lfloor \textrm{gcd}(g-k,k^2-1)/(k + 1)\rfloor =m-1$. We also extend his study of cyclic Young graphs and prove a minor result on isomorphism and the nodes adjacent to the node [0, 0]. (2015, p. 1)

Besides these studies on reverse multiples, Benjamin Holt’s work should also be mentioned. In a series of three papers (2014; 2016; 2017) that have all been published in the electronic journal *Integers*, he also investigates the general phenomenon of reverse multiples. In (Holt 2014), he establishes some general properties of reverse multiples of any base with an arbitrary number of digits using only elementary methods. In (2016), Holt builds on Sloane and Kendrick’s work and is concerned with several families of *derived* reverse multiples, i.e., reverse multiples where the carry numbers themselves are digits of lower-base reverse multiples. Finally, in (Holt 2017), he generalizes results on reverse multiples to an arbitrary permutation, i.e., he investigates numbers which are integer multiples of some permutation of their digits, which he calls *permutibles*.

## Hardy on *Serious* Mathematics

As mentioned earlier, Hardy’s *Apology* is meant to provide a justification of the study of mathematics and that of a professional mathematician’s life, in particular that of his own. He thinks that the “real” mathematics, i.e., the mathematics of the best mathematicians (pure or applied), such as Fermat, Euler, Gauss, Abel and Riemann (p. 119)^4^, but also Maxwell, Einstein, Eddington and Dirac (p. 131), is “almost wholly ‘useless’” (p. 119), so that one cannot justify it by its practical utility. According to Hardy, the real mathematics must be justified as (a creative) art.

I have already pointed out in the introduction that Hardy introduces examples (*a*) and (*b*) in his *Apology* as examples of *non-serious* mathematical theorems. They appear together with his diagnosis $(\mathcal {D})$ in § 15, where he starts to explain a little more precisely what he means by a “serious” theorem and a “significant” mathematical idea. In the remainder of this section, I present Hardy’s main thoughts on these qualities which he sets forth in his *Apology*.

The first time Hardy mentions the *seriousness* of a theorem and the *significance* of a mathematical idea is in § 11, where he explains quite at the beginning that contrary to chess problems which are “genuine mathematics,” but “in some way ‘trivial’ mathematics,” since “there is something essential lacking,” namely importance, “[t]he best mathematics is *serious* as well as beautiful—‘important’ if you like, but the word is very ambiguous, and ‘serious’ expresses what I mean much better” (pp. 88f.). To further illustrate his ideas, he says that (hereinafter referred to as (*S1*))

(*S1*): The ‘seriousness’ of a mathematical theorem lies, not in its practical consequences, which are usually negligible, but in the *significance* of the mathematical ideas which it connects. We may say, roughly, that a mathematical idea is ‘significant’ if it can be connected, in a natural and illuminating way, with a large complex of other mathematical ideas. Thus a serious mathematical theorem, a theorem which connects significant ideas, is likely to lead to important advances in mathematics itself and even in other sciences. No chess problem has ever affected the general development of scientific thought; Pythagoras, Newton, Einstein have in their times changed its whole direction. (pp. 89f.)

He continues by emphasizing that “[t]he seriousness of a theorem, of course, does not *lie in* its consequences, which are merely the *evidence* for its seriousness” (p. 90).

He then goes on and gives some examples of serious mathematical theorems. He also provides proofs for two of them, namely Euclid’s proof that there are infinitely many prime numbers (§ 12) and Pythagoras’s proof that $\sqrt{2}$ is irrational (§ 13). He concludes these two sections by pointing out that compared to “Dudeney’s most ingenious puzzles” or “the finest chess problems that masters of that art have composed,” the superiority of both Euclid’s theorem and that of Pythagoras stands out and that “there is an unmistakable difference of class” (p. 98). The two ways in which their superiority stands out is in their *beauty* and their *seriousness*. In § 14, he then starts to discuss the latter notion in more detail. There he says that this superiority is “obvious and overwhelming” (p. 99) and explains that (hereinafter referred to as (*S2*))

(*S2*): The chess problem is the product of an ingenious but very limited complex of ideas, which do not differ from one another very fundamentally and have no external repercussions. We should think in the same way if chess had never been invented, whereas the theorems of Euclid and Pythagoras have influenced thought profoundly, even outside mathematics. (ibid.)

With respect to Pythagoras’s proof, Hardy points out that it is

capable of far-reaching extension, and can be applied, with little change of principle, to very wide classes of ‘irrationals’. We can prove very similarly (as Theodorus seems to have done) that

$$\begin{aligned} \sqrt{3},\,\sqrt{5},\,\sqrt{7},\,\sqrt{11},\,\sqrt{13},\,\sqrt{17} \end{aligned}$$

are irrational, or (going beyond Theodorus) that $\root 3 \of {2}$ and $\root 3 \of {17}$ are irrational. (p. 100)

In general, he emphasizes the importance of both theorems in the development of (modern) mathematics. While “Euclid’s theorem is vital for the whole structure of arithmetic” (p. 99), “Pythagoras’s theorem and its extensions tell us that, when we have constructed this arithmetic, it will not prove sufficient for our needs, since there will be many magnitudes which obtrude themselves upon our attention and which it will be unable to measure” (p. 100).

Hardy begins § 15 by reiterating that “[a] ‘serious’ theorem is a theorem which contains ‘significant’ ideas” (p. 103). Since he thinks that “[w]e can recognize a ‘significant’ idea when we see it,” he tries to give “some sort of analysis” (ibid.). He mentions “two things at any rate which seem essential” to a significant idea, namely “a certain *generality* and a certain *depth*” (ibid.). With respect to the notion of generality, he gives the following, tentative characterization:

**Characterization of** ***Generality***: “A significant mathematical idea, a serious mathematical theorem, should be ‘general’ in some such sense as this.”

(*G1*): “The idea should be one which is a constituent in many mathematical constructs, which is used in the proof of theorems of many different kinds.”
(*G2*): “The theorem should be one which, even if stated originally (like Pythagoras’s theorem) in a quite special form, is capable of considerable extension and is typical of a whole class of theorems of its kind.”
(*G3*): “The relations revealed by the proof should be such as connect many different mathematical ideas.” (p. 104)^5^

I speak of a “tentative” characterization, since immediately after he has presented the ideas above, Hardy states that “[a]ll this is very vague, and subject to many reservations” (ibid.). He continues, however, as follows (hereinafter referred to as $(\mathcal {A})$, where “$\mathcal {A}$” is meant to remind of “assessment”):

$$(\mathcal {A})$$
But it is easy enough to see that a theorem is unlikely to be serious when it lacks these qualities conspicuously; we have only to take examples from the isolated curiosities in which arithmetic abounds. I take two, almost at random, from Rouse Ball’s *Mathematical Recreations*. (ibid.)

He then presents his two examples (*a*) and (*b*), immediately followed by his diagnosis $(\mathcal {D})$ which closes § 15.

In § 16, Hardy discusses the fact that in some sense, all mathematics is equally general, or *abstract*, which should not be confused with his notion introduced in his previous section. As he emphasizes, “[w]e are looking for *differences* of generality between one mathematical theorem and another” (p. 107).

Finally, in § 17, he addresses the notion of *depth*, which a significant idea should have in addition to its generality. The notion of depth, however, “is still more difficult to define” (p. 109) and is an elusive one even for mathematicians capable of recognizing it (p. 112). He explains that “[i]t has *something* to do with *difficulty*; the ‘deeper’ ideas are usually the harder to grasp: but it is not at all the same” (p. 109). Presumably as a kind of first approximation, Hardy states:

It seems that mathematical ideas are arranged somehow in strata, the ideas in each stratum being linked by a complex of relations both among themselves and with those above and below. The lower the stratum, the deeper (and in general the more difficult) the idea. (p. 110)

This is why he considers the “idea of an ‘irrational’” to be “deeper than that of an integer”; and, because of that, Pythagoras’s theorem as deeper than Euclid’s (ibid.). Moreover, he regards Euclid’s theorem to be “very important, but not very deep” since the deepest notion involved in the proof is that of divisibility (p. 111).

Taking into account the research that has been done on the general phenomenon of reverse multiples, which I discussed at least in part in Sect. 2, I would now like to take a closer look at Hardy’s assessments of this phenomenon, which he makes in $(\mathcal {A})$ and $(\mathcal {D})$, in terms of their accuracy.

## A Closer Look at Hardy’s Notion of *Generality*

We will now begin with evaluating Hardy’s comments about his example (*a*). In this regard, we will first discuss his assessment $(\mathcal {A})$ in which he addresses his qualities (*G1*) to (*G3*). After that, I will identify and argue for what I think is the most important aspect of Hardy’s notion of generality. This will be crucial to my evaluation of Hardy’s diagnosis $(\mathcal {D})$, which I will turn to in Sect. 5.

### Example (a) and the Three Qualities of *Generality*

Let us now take a closer look at Hardy’s assessment of example (*a*) made in $(\mathcal {A})$ in which he refers to the statements (*G1*)–(*G3*), by going through all these properties one by one (in reverse order). Although it is not easy to evaluate his assessment—not least because all the qualities he proposes in his characterization are “very vague, and subject to many reservations”—I think it is worth doing anyway, partly because it will help bring some clarity to what I think is the most important aspect of *generality*. In assessing his statements, we should also keep in mind the context in which they appear in his *Apology*: Hardy tries to characterize the *best* mathematics, which is both beautiful *and* serious. In this regard, I will compare the respective findings with his two most elaborated examples of serious theorems, namely those of Pythagoras and Euclid.

Although Hardy does not give a specific proof of example (*a*) in his *Apology*, he must have had a rather straightforward one in mind. A proof in this category can be found, for instance, in (Webster and Williams, 2012/2013). To avoid some repetitive work, one can first establish the fact that in base 10, the only possible values for *k* are 4 and 9. A proof of this must not be restricted to the four-digit case, since it can be essentially the same for an arbitrary number of digits—as the one proposed by Webster and Williams. For all the other possible values for *k*, i.e., 2, 3, 5, 6, 7 or 8, one can arrive at contradictions with the help of the observation that $ka_{n-1}$ must be less than or equal to 9 (to retain the number of digits) and (in the more “difficult” cases) by comparing possible values for $a_{n-1}$ and $a_0$. After this, one can convince oneself that there are no 4 and 9-reverse multiples with less than four digits by obtaining some simple contradictions and similarly that 1089 and 2178 are *the only* two four-digit reverse multiples.

Now, if we admit that Hardy is right in $(\mathcal {A})$ that example (*a*) lacks quality (*G3*) conspicuously, one might wonder whether his two examples from Greek mathematics do not also lack this quality, especially since, as Hardy himself points out, “[t]hey are ‘simple’ theorems, simple both in idea and in execution” (p. 92).

With respect to (*G2*), possible examples of theorems of the kind of example (*a*) are theorems of the form

- *a*, *b*, *c*, ...are the only *n*-digit reverse multiples in base *g*;
- there are only *m* *k*-reverse multiples in base *g* with less than *n* digits;
- all *k*-reverse multiples in base *g* are of the form $ABCD\dots EFGH$ (see, for instance, Theorem 2.10 in (Webster and Williams, 2012/2013));
- the generating function for (*g*, *k*)-reverse multiples is given by $\mathcal {C}(x)=\sum _{t\ge 0}c_tx^t$;
- the (*g*, *k*) Young graph is isomorphic to the $(g',k')$ Young graph;
- the digits of the reverse multiple *x* of the family $\mathcal {F}$ of reverse multiples correspond to the carry numbers of the higher-base reverse multiple *y*;
- etc.

While I do think that Hardy was well aware that one could produce countless theorems of the form listed in the first two to three points above (that are not only concerned with the base-10 case)—which is why he probably added the restriction “considerable” to (*G2*)—I nevertheless think that he might have underestimated the potential of (*a*) with respect to (*G2*), given the other items of the enumeration.

Besides the particular examples of irrationals to which Pythagoras’s proof can be applied “with little change of principle” which I have reproduced in Sect. 3, Hardy also refers in a subsequent footnote to Chapter IV of *An Introduction to the Theory of Numbers* (1938) which he co-authored with E. M. Wright “where there are discussions of different generalizations of Pythagoras’s argument” (p. 100). More precisely, Hardy and Wright present two proofs of the theorem that

$\root m \of {N}$* is irrational, unless N is the m*-*th power of an integer* *n*.

one of which is a (close) generalization of Pythagoras’s proof that Hardy provides in the *Apology*, and one proof of the theorem that

If *x is a root of an equation* $x^m+c_1x^{m-1}+\ldots +c_m=0$, *with integral coefficients of which the first is unity, then* *x* *is either integral or irrational.*

which is a generalization of a “very similar” argument to that of Pythagoras (1938, pp. 39–41).

However, compared to these extensions of Pythagoras’s proof or to the case of Euclid’s theorem, where Hardy mentions no extension at all, it seems that (*a*) is not worse off.

To sum up, I take it to be the case that example (*a*) is not (decisively) worse off than Hardy’s two serious theorems with respect to the qualities (*G3*) and (*G2*).

That being said, I think this is quite different for quality (*G1*). Although again he does not elaborate on how exactly example (*a*) conspicuously lacks this quality, I think that “the idea” he speaks of in (*G1*) refers in this case to the concept of a reverse multiple itself.^6^

First, while there are—as we saw in Sect. 2—some possible “constructs” in which this idea is a constituent, such as Ludington Young’s rooted trees, Sloane’s Young graphs, and Holt’s permutibles, I do not think there are enough to count as “many” (as required by (*G1*)). Secondly, and, what I take to be much more important, is the fact that the idea of a reverse multiple—even considering all the research that has been carried out on this phenomenon since the publication of Hardy’s *Apology*—does not (yet) occur in proofs of theorems of *many different kinds*. For instance, (so far) Young graphs do not appear in proofs of theorems of other areas of research.

This is in stark contrast to Hardy’s two main examples of serious theorems. Because of the fundamental character of the ideas contained in the theorems of Euclid and Pythagoras for the development of mathematics, namely the idea of the infinity of primes or the notion of a prime number itself and the idea of an irrational number, it is clear that they score highly in terms of quality (*G1*).

### The Most Important Aspect of *Generality*

We should now take a step back and have a look at qualities (*G1*)–(*G3*) themselves and how they relate to each other. While quality (*G1*) is explicitly about the generality of mathematical ideas themselves, (*G2*) addresses theorems in general and (*G3*) their proofs.

When comparing these qualities of generality—which itself is introduced as an essential property of a significant mathematical idea and a serious theorem—we find that a general theme present in all of them is the connectedness with more mathematics. As the reader may recall (cf. especially (*S1*)), according to Hardy, the seriousness of a mathematical theorem lies in the significance of the ideas which it connects or contains, which makes quality (*G1*) the most fundamental and important one out of the three, even if one is interested in the generality (and ultimately in the seriousness) of a theorem and its proof. This is also immanent in the discussion of Sect. 4.1 above, for as we have seen, it is precisely this quality that distinguishes the serious (and thus general(!)) theorems of Euclid and Pythagoras.

As a reminder, in Hardy’s first, rough characterization of *significance* (cf. (*S1*)), he explains that a mathematical idea is significant “if it can be connected, in a natural and illuminating way, with a large complex of other mathematical ideas.” I think that part of this characterization is that these “other mathematical ideas” should also be distinctly *different* from each other in a large number. This is supported, first and most importantly, by the fundamental quality (*G1*) itself, where he explicitly talks about “many *different* kinds” (emphasis added) (and for that matter also by (*G3*), where the emphasis is on a connectedness to many different mathematical ideas). Secondly, indirectly by his comparison of serious mathematical theorems with chess problems: recall that in § 14 of the *Apology*, Hardy discusses the seriousness of Euclid’s theorem and that of Pythagoras as one important way in which their superiority lies when compared to chess problems. There, he emphasizes that a chess problem “is the product of an ingenious but very limited complex of ideas, which do not differ from one another very fundamentally and have no external repercussion” (cf. (*S2*)). Finally, I think that the aspect of connectedness to other, different mathematics is also indirectly emphasized in $(\mathcal {A})$, when Hardy uses its antonym to refer to his examples (*a*) and (*b*) by calling them *isolated* curiosities—especially since I am convinced that in the case of (*a*) he was well aware that one could produce innumerable theorems of the form given in the first two items of my list presented in Sect. 4.1.

For these reasons, I believe that *(nontrivial) connectedness to a large complex of appreciably different mathematical ideas* is not only the most important aspect of Hardy’s notion of generality, but also a necessary condition for an idea to be considered significant or a theorem to be serious.

Before moving on to the next section where we will have a look at Hardy’s diagnosis here referred to as $(\mathcal {D})$, there are three points which should be made.

First, I have added the term “nontrivial” to my characterization of the most important aspect of generality (at least in parentheses) to do justice to the fact that qualities (*G1*)–(*G3*) do not talk about *any* connectedness to other mathematical ideas, but about quite specific ones. For instance, given the foundational role of set theory, essentially all mathematical concepts and results can be formalized within this theory, thus establishing a connection between all of them. However, if this was the apparent only connection between two different concepts, it would be quite trivial in some respects.^7^

Secondly, one should notice that, according to my conclusion, scoring well on quality (*G2*) while lacking the other two, makes a theorem not general, at least not in the sense required for it being serious. I think this is a welcome result, since a rather isolated mathematical theorem is not made much less isolated, at least not if it is understood as part of the broad mathematical research landscape, when only other results *of its kind* are added. Moreover, it seems that such a theorem is very unlikely to lead to important advances in mathematics, unlike serious theorems (cf. (*S1*)).

Thirdly, while I believe that Hardy admits that the seriousness of a theorem and the significance of a mathematical idea can come in degrees, I do not think that this compromises the correctness of my conclusion above, especially with respect to its second part that the most important aspect I have identified is also a *necessary condition* for significance and seriousness. To begin with, that these notions can actually come in degrees is in my opinion contained in Hardy’s conclusion of the *Apology*. While he focuses on examples of the *best* mathematics in his discussion of the seriousness of a theorem, in his ultimate conclusion on the value of his own mathematical life he declares that his work differs only in degree, *not in kind*, from that of the great mathematicians:

The case for my life, then, or for that of any one else who has been a mathematician in the same sense in which I have been one, is this: that I have added something to knowledge, and helped others to add more; and that these somethings have a value which differs in degree only, and not in kind, from that of the creations of the great mathematicians, or of any of the other artists, great or small, who have left some kind of memorial behind them. (p. 151)

That he does not think, however, that all mathematics is of this kind with respect to seriousness is shown, among other things, by his demarcation of chess problems, which are “genuine mathematics,” *but lack something essential*, namely importance or seriousness, and “real” mathematics (cf. Sect. 3); and by his examples (*a*) and (*b*) “in which arithmetic abounds” themselves *as proposed examples* of mathematical(!) theorems *which are not serious*.^8^ I think my discussion above makes it perfectly reasonable to call theorems—within Hardy’s framework—not of that *kind* which are conspicuously lacking in the most important aspect of generality, as I have identified it.

## Hardy’s Diagnosis Revisited

As a reminder, in § 15–§ 17 of the *Apology* (cf. Sect. 3), Hardy explains a little more precisely what makes a mathematical idea *significant*, or a theorem *serious*. Specifically, in § 15, he focuses on the notion of generality in the sense that this quality is essential to a serious theorem or a significant idea. After having introduced his tentative characterization of generality consisting itself of the qualities (*G1*)–(*G3*), he presents (*a*) and (*b*) as examples which are said to be conspicuously lacking in these qualities (cf. $(\mathcal {A})$). He then concludes the section with diagnosis $(\mathcal {D})$.

### Example (a) and its Generalizations

Let us first evaluate the second part of $(\mathcal {D})$, namely Hardy’s statement that “[t]he theorems are not serious; and it is plain that one reason (though perhaps not the most important) is the extreme speciality of both the enunciations and the proofs, which are not capable of any significant generalization,” with respect to example (*a*).

To begin with, I just want to take a quick look at Hardy’s insertion that the reason he gives is “perhaps not the most important” one, because he does not further elaborate on this. As mentioned in Sect. 3, for Hardy a certain generality is not the only thing which seems essential for a significant idea. The other one he mentions in § 15 and elaborates in § 17 is depth. So perhaps he meant to imply that the main reason why the examples (*a*) and (*b*) are not serious is that they are not deep. However, I will refrain from any further speculations on this issue and turn to the more interesting part of his statement, at least with regards to my concerns here.

First, I think that we should not confuse his use of the word “significant” in this sentence with his rather technical notion of *significance*, which he uses in the context of a “significant idea.” This is because at this point in the *Apology* he has only begun a more detailed analysis of his technical notions of significance and seriousness, which will occupy him up to and including § 17. A reference to this notion in the conclusion of § 15, i.e., in the last sentence of $(\mathcal {D})$, would then in principle transform it into a statement such as “These are not serious theorems, because they are not serious.”

Moreover, when we try to make sense of this sentence, we must of course consider the context in which it appears. It is the last sentence of § 15—in which Hardy is mainly concerned with the notion of generality—and should be understood as such, namely as his concluding remark about why examples (*a*) and (*b*) are not serious, given his assessment of their generality. While he has already stated in $(\mathcal {A})$ that these examples are conspicuously lacking in qualities (*G1*)–(*G3*), I think he added the phrase “which are not capable of any *significant* generalization” (emphasis added) to emphasize that there are also no generalizations of (*a*) and (*b*) that would qualify as “general” themselves—at least not in the sense or to the extent necessary to count as significant ideas or serious theorems themselves. In other words, he wanted to make clear that not only examples (*a*) and (*b*) show an extreme speciality in both their enunciations and their proofs, but also that there are no generalizations which would make them much less isolated, to the point where one might start doubting whether his two examples are in fact examples of non-serious theorems.

It is necessary to stress this point, since I think it has been misinterpreted by several of the researchers who have recently contributed to the general phenomenon of reverse multiples. So, before I will evaluate whether Hardy’s upshot is still tenable after all the research that has been carried out on this phenomenon, let us first have a look at what some of the researchers have to say about this.

For instance, Lara Pudwell (2007, p. 129) states that

In *A Mathematician’s Apology* [(Hardy 1993); S.W.] G. H. Hardy states, “8712 and 9801 are the only four-figure numbers which are integral multiples of their reversals”; and, he further comments that “this is not a serious theorem, as it is not capable of any significant generalization.” However, Hardy’s comment may have been short-sighted. In 1966, A. Sutcliffe [(1966); S.W.] expanded this obscure fact about reversals. Instead of restricting his study to base 10 integers and their reversals, Sutcliffe generalized the problem to study all integer solutions of

$$\begin{aligned} k(a_hn^h+a_{h-1}n^{h-1}+\cdots +a_1n+a_0)=a_0n^h+a_1n^{h-1}+\cdots +a_{h-1}n+a_h \end{aligned}$$

with $n\ge 2$, $1<k<n$, $0\le a_i\le n-1$ for all *i*, $a_0\ne 0$, $a_h\ne 0$.

Benjamin Holt (2014, pp. 1f.) claims that

The most well-known examples of base-10 palintiples include 87912 and 98901 since $87912 = 4\cdot 21978$ and $98901 = 9\cdot 10989$. [...] At first glance it may seem that such numbers are merely curiosities that only make for cute puzzle problems. Such was the belief of G. H. Hardy who, in his classic essay *A Mathematician’s Apology* [(Hardy 1993); S.W.], cited the fact that “8712 and 9801 are the only four-figure numbers which are integral multiples of their ‘reversals’” as an example of a theorem that is not “serious.” Furthermore, “[this fact is] very suitable for puzzle columns and likely to amuse amateurs, but there is nothing in them which appeals much to a mathematician” and is “not capable of any significant generalization.” Sutcliffe [(1966); S.W.], Pudwell [(2007); S.W.], and Young [(Ludington Young 1992a); S.W.] demonstrate otherwise; in spite of Hardy’s comments the palintiple problem generalizes quite naturally.

And Lev Kendrick (2015, pp. 1f.) says that

Ironically, the most prominent mention of these numbers may well have curtailed their prominence: G. H. Hardy, in *A Mathematician’s Apology*, refers to the fact that 1089 and 2178 are the only four-digit (10, *k*) reverse multiples as one “very suitable for puzzle columns and likely to amuse amateurs,” but “not serious” and “not capable of any significant generalization.” While we abstain from judgment on the first two counts, it is clear, after the intervening decades, that Hardy misjudged regarding the third. [footnote: The only refuge for his position being in the nebulous qualifier “significant.”] A number of works generalize the problem, a list of most of which may be found in Sloane’s 2013 paper [(2014); S.W.] on the topic [cf. Sloane’s comment below; S.W.]; [...].

Despite what these researchers say, I do not think that the work done so far on reverse multiples, and especially not the papers explicitly or, in Kendrick’s case, implicitly mentioned by them (and by Sloane in his comment below), namely (Sutcliffe 1966), (Pudwell 2007) and (Ludington Young 1992a), show that Hardy was wrong. In general, I believe their diagnoses are ultimately due to a misinterpretation of the “nebulous qualifier ‘significant’” (as Kendrick puts it).

As I have already explained above, the context in which diagnosis $(\mathcal {D})$ appears in the *Apology*—and hence the phrase “which are not capable of any significant generalization”—suggests that this qualifier refers to Hardy’s assessment of the generality of the “isolated curiosities” (*a*) and (*b*), and to whether possible generalizations would call into question their (alleged) status of being isolated. I have argued in Sect. 4.2 that the most important aspect of generality and at the same time a necessary condition for an idea to be considered significant or a theorem to be serious is the (nontrivial) connectedness to a large complex of appreciably different mathematical ideas. Although the research done so far has shown that the general phenomenon of reverse multiples is anything but trivial (in terms of difficulty), I do not think it has shown that this phenomenon is connected to so much other, different mathematics that it would no longer be considered “isolated.” In particular, in the case of Ludington Young’s (1992a) work, presented in the comments by the researchers above as a counterexample to Hardy’s claim, the reader can easily see for themselves, as I discussed it quite extensively in Sect. 2, that while Ludington Young has introduced a new technique (in the form of graphical representations) for studying reverse multiples, this technique does not in itself show that this phenomenon is connected to a much larger complex of other, *different* mathematical ideas, which would make it much less isolated. Moreover, as I have already mentioned in Sect. 4.1 in the context of evaluating example (*a*) with respect to quality (*G1*), even the follow-up work on these graphical representations in form of Sloane’s Young graphs has not (yet) shown that the phenomenon of a reverse multiple is (nontrivially) connected to other areas of research, since these graphs do not (yet) appear in proofs of theorems of different kinds.

In my opinion, Neil Sloane has misinterpreted the phrase “not capable of any significant generalization” in a similar way to Pudwell, Holt, and Kendrick. But in doing so, he also presents another interpretation of the entire passage. He says (2014, p. 99):

In 1940, G. H. Hardy [(Hardy 2000); S.W.] famously remarked that the existence of these two numbers was “likely to amuse amateurs”, but was not of interest to mathematicians, since this result is “not capable of any significant generalization”. It seems fair to say that Hardy was wrong, since references [(Grimm and Ballew 1975), (Laisant et al. 1908), (Kaczynski 1968), (Klosinski and Smolarski 1969), (Pudwell 2007), (Sutcliffe 1966), (Webster and Williams 2012/2013), (Ludington Young 1992a), (Ludington Young 1992b); S.W.] discuss generalizations.^9^

Similarly, the mathematician Solomon Golomb (2014) explains in his review of Sloane’s article that

Using the special case of the two numbers 1089 and 2178, G. H. Hardy gave this problem notoriety in his *A mathematician’s apology* [(Hardy, 1940); S.W.], by commenting that it was “likely to amuse amateurs” but was not of interest to mathematicians, since it was “not capable of any significant generalization”. With the present paper, there are now at least ten works that discuss nontrivial generalizations. This furnishes yet another example (along with his statement that relativity and quantum mechanics were unlikely to have practical applications) of the unreliability of Hardy’s assertions in his famous *Apology*.

However, in $(\mathcal {D})$, Hardy is only using the phrase “not capable of any significant generalization” in his upshot of why examples (*a*) and (*b*) are *not serious*. While Hardy might have been of the opinion that (*a*) is ultimately not of interest to mathematicians because it is not capable of any significant generalization, this is not what he says in $(\mathcal {D})$. In this context, one might also wonder whether Hardy thought that only serious theorems are, or rather *should be* of interest to a (real) mathematician. Especially the final conclusion of his *Apology* (cf. the end of Sect. 4.2), which occurs in the context of his justification of his own mathematical life, makes such a normative view on Hardy’s part not implausible. Although I will not go into this any further here, I will return to mathematicians’ interests more generally in Sect. 5.2.

Overall, while I do not want to completely rule out the possibility that at some point it might turn out that reverse multiples can be (nontrivially) connected with a large complex of different mathematics, I still think Hardy’s conclusion, expressed in the last sentence of diagnosis $(\mathcal {D})$ is tenable.

Note that some (if not all) of the above comments by researchers, especially those of Pudwell and Holt, seem to suggest that Hardy was not aware that his base-10 example (*a*) can also be generalized to arbitrary bases and that one can also seek solutions to the general Equation (1). However, this assumption does not seem very convincing to me. Why should he not have been aware of this obvious, natural idea? In this respect, I think my interpretation, together with my evaluation of Hardy’s assessment of (*a*) with respect to (*G2*) (cf. Sect. 4.1) is much more plausible.

### Mathematical Interest is Multifaceted

In the first sentence of $(\mathcal {D})$, Hardy states that examples (*a*) and (*b*) “are likely to amuse amateurs, but there is nothing in them which appeals much to a mathematician.” What about the accuracy of this statement in light of the research that has been conducted on the general phenomenon of reverse multiples since the publication of Hardy’s *Apology*?

Section 2 shows that quite a few researchers are/were interested in reverse multiples over the last sixty years or so. While some of them might qualify as “amateur mathematicians”—meaning that they have not done much other, more traditional work at the university research level—and in this sense maybe qualify as “amateurs” in Hardy’s understanding, such as Sutcliffe, Holt and Kendrick,^10^ this does not apply to everyone. For example, not to Lara Pudwell, who, although still a doctoral student at the time of publication of her (2007), is now a full professor in the department of mathematics and statistics at Valparaiso University; and not to Anne Ludington Young, who is an emeritus professor of mathematics at Loyola University Maryland; and, of course, not to the renowned mathematician Neil Sloane. At least these examples suggest that, although, as Kendrick appropriately points out, Sloane’s list of references “is rather short for a problem given such exposure as a mention in Hardy’s book” (2015, p. 2), Hardy’s diagnosis that there is nothing in example (*a*) “which appeals much to a mathematician” is not true in all its generality. Concerning the “appeal” of some of the research carried out on reverse multiples, Sloane makes some interesting comments to which we will now turn.

After having determined the generating function for the number of all base-10 reverse multiples, regardless of the multiplier (Equation (6)), Sloane (2014, p. 109) asks: “Would this generating function have changed Hardy’s opinion of the problem?,” which, most likely, is meant to mainly address the appeal of example (*a*) for Hardy, based on what Sloane said in a talk about this topic. In this talk which he gave at the *Rutgers Experimental Mathematics Seminar* at Rutgers University on October 10, 2013, this question also appeared on his slides after he has determined the generating function (6). Although he did not read it out loud, he said with respect to his result “This might even have impressed Hardy” but added immediately after “Though I doubt it” (2013, 22:18–22:22).

Furthermore, Sloane explains in the abstract of his article: “These Young graphs are *interesting* finite directed graphs, whose structure is not at all well understood” (2014, p. 99, emphasis added). And, in his talk, he calls Ludington Young’s (1992a; b) “two really interesting papers” (2013, 6:10–6:18).

These are all statements in which Sloane expresses his own appreciation of (some results on) the phenomenon of reverse multiples.

We could even go a step further and argue with the help of Robert Thomas’s (2017), that the articles mentioned in Sect. 2, because they all have been published in journals, already show value, namely the aesthetic value of being interesting. As Thomas (2017, p. 118) claims—mainly based on his ten years of experience as a managing editor of a mathematical research journal—“‘interesting’ is a *sine qua non* of publishable mathematical research” and, consequently, that it “is an aesthetic feature seen in all published mathematics.”

In this context, one might wonder, however, whether one should treat all publications in the same way. In mathematics, there is an (alleged) distinction that is occasionally brought up, namely that between serious and *recreational* mathematics, which is perhaps also partly reflected in Hardy’s discussion. As a reminder, examples (*a*) and (*b*) were taken, by Hardy, “almost at random, from Rouse Ball’s *Mathematical Recreations*” (cf. $(\mathcal {A})$). So, if a paper, such as Grimm and Ballew’s (1975) is published in a journal devoted to recreational mathematics, is then its aesthetic feature of being interesting of the same kind as a paper published in journals exclusively devoted to “serious mathematics”? Or should not also all the other work done on reverse multiples be characterized as rather recreational? Furthermore, is there a general difference *in kinds* between interesting serious and interesting recreational mathematics?

The problem with these questions is that there is no sharp boundary between “serious” and “recreational” mathematics. For instance, a problem may start out as a mere recreation, but develop into quite useful mathematics. As Ian Hacking (2014, p. 77) explains:

There are paths that lead on from mere amusements to things that Hardy might count as serious. The example of squaring the square is a case in point. Tutte’s own (1958) account of the events was published in Gardner’s column. The problem began in part with Dudeny’s puzzle of Lady Isabel’s Casket (§1.28). It turned into moderately deep questions with applications to electrical analysis. And Hardy’s collaborator Littlewood used the proof, of the impossibility of a cube dissection, as an exemplar of good proof. A silly puzzle evolves into fairly good maths.

There would be much more to say about the merits of recreational mathematics and its connection to more “serious” mathematics, but that is beyond the scope of this article. Let us conclude this section by returning once again to Neil Sloane.

As for him, he is not much concerned with such a distinction. In an interview published in *Quanta Magazine* (Klarreich 2015), Sloane was asked whether he felt “that there is a divide between ‘serious mathematics’ and ‘recreational mathematics’,” or whether he tended “not to think in those terms” to which he answered:

I don’t think in those terms. I don’t think there’s much difference. If you look hard enough, you can find interesting mathematics anywhere. (ibid.)

Therefore, I think Sloane’s disagreement with Hardy on the appeal of reverse multiples to a mathematician is a genuine example of the multifaceted nature of mathematical interest.

## Conclusion

As we have seen, some work has been done on the general phenomenon of reverse multiples since the publication of Hardy’s *Apology*. Among other things, particular finite, directed graphs, called Young graphs, were introduced to study this phenomenon.

I have argued, however, that this work does not (yet) threaten Hardy’s claim that his example (*a*) is “not capable of any significant generalization.” To do this, I have first argued that when read in its context, this phrase should be understood as an emphasis that there are no generalizations of this example (and of example (*b*)) that would qualify as “general” themselves, at least not in the sense or to the extent necessary to qualify as candidates for serious theorems. Then, with the help of what I take to be the most important aspect of Hardy’s notion of generality and a necessary condition for an idea to be considered significant or a theorem to be serious, namely its *(nontrivial) connectedness to a large complex of appreciably different mathematical ideas*, I have argued that Hardy’s upshot is still tenable, which is in contrast to what several researchers, who worked on the general phenomenon of reverse multiples, say.

Finally, I have suggested that especially Sloane’s appreciation of some results on reverse multiples, in contrast to Hardy’s generally dismissive attitude towards the notion of a reverse multiple, makes this phenomenon a genuine example of the multifaceted nature of mathematical interest.

## Footnotes

1 “Quels sont alors les nombres *n* plus petits que la base du système, tels qu’il existe des produits *n*N qui soient les renversés de N?”

2 Note that the letters that I have used in the statement of the general equation in which Sutcliffe is interested in, have been changed to be consistent with (Ludington Young 1992a, b) and (Sloane 2014).

3 Sloane (2014, p. 100) states in the introduction that “the first mention of this fact appears to have been by D. W. Wilson” in his comment in OEIS on the sequence A001232 (1997). However, already in a talk presented at a meeting of the *Berliner Mathematischen Gesellschaft* in 1915, the member Robert Burg made the connection between the numbers of base-10 reverse multiples and the Fibonacci numbers (Burg 1916, pp. 15f.). There, Burg also noticed that (10, 9)-reverse multiples are precisely the numbers $\gamma \cdot \beta$, where $\gamma =99$ and $\beta$ is any positive palindromic decimal number consisting only of the digits 1 and 0, but where a single 1 or 0 may never occur (1916, p. 18). This fact is again attributed by Sloane (2014, p. 100) to Wilson (1997).

4 Page references without additional information in the remainder of this article always refer to (Hardy, 1940/2012).

5 Note that Hardy does not present this within a “characterization” environment in the original text, along with an enumeration (*G1*)–(*G3*). I have introduced this for ease of reference in my later discussion. In the *Apology* the individual sentences that appear in my “characterization” simply occur as a continuous text.

6 If this seems obvious to the reader, so much the better for my argumentation. There are, however, some complications in trying to evaluate his assessment made in $(\mathcal {A})$ in terms of the quality (*G1*) in thinking about example (*a*). As we now know, for Hardy a serious theorem is one that *connects* or *contains* significant ideas. The question that arises in this context is whether we should treat theorems and their proofs separately. The passage of the *Apology* in which Hardy discusses the “purely aesthetic” qualities of the theorems of Euclid and Pythagoras seems to contradict such an interpretation. There, indeed, the theorems and proofs are considered as a kind of unity. As Hardy puts it: “In both theorems (and in the theorems, of course, I include the proofs) there is a very high degree of *unexpectedness*, combined with *inevitability* and *economy*” (p. 113). The same approach of considering theorems *together* with their proofs is also inherent in his discussion of why Euclid’s theorem is important, *but not very deep* (cf. the end of Sect. 3). In fact, (*G3*) itself indicates that a proof of a theorem can also play a role in evaluating theorems in terms of their seriousness. In general, we might wonder whether a theorem, which is about rather “non-general” (with respect to (*G1*)), “superficial” or non-significant ideas, but which requires a highly nontrivial proof that involves a huge amount of significant ideas might nevertheless turn out to be serious. In this context, one might also wonder whether different proofs of a theorem have different effects on the seriousness of a theorem. With respect to the former question, a possible indication that it would have been answered by Hardy in the negative can be found in his discussion of the notion of depth where he compares this notion to *difficulty* and explains that there are not only cases of deep theorems which are not difficult, but also those which “may be essentially superficial and yet quite difficult to prove” (p. 110). Nevertheless, we cannot answer these questions satisfactorily with what Hardy gives us in his *Apology*.

7 Note that part of Hardy’s first, rough characterization of the *significance* of a mathematical idea is not only its connectedness to a large complex of other mathematical ideas, but also *the way* these other ideas are connected to the one, particular idea. They are supposed to be connected in a *natural and illuminating* way. The need of adding this condition seems to be in line with the example of set theory as a foundation for mathematics above. (Thanks to an anonymous referee for prompting me to address this point.) One might wonder, however, in what way this additional condition relates to Hardy’s discussions of generality and depth in § 15 and § 17, respectively. Perhaps this condition is (at least partially) implicitly addressed in these discussions; or it should even be seen as a further condition for an idea to be considered significant in the first place. Unfortunately, Hardy does not address this issue. And since I am mainly concerned in this article with his discussion of generality presented in § 15, I will not speculate further on it here.

8 Note that Hardy’s distinction between real mathematics and “trivial” mathematics which he addresses in § 25–§ 28 is primarily based on their “aesthetic merit” (p. 134), i.e., on the second criterion, besides seriousness, by which the patterns of mathematicians ought to be judged.

9 Note that two of the papers listed by Sloane have not been mentioned in Sect 2. Both of them are rather short papers, not longer than three pages. After characterizing all base-10 reverse multiples, Klosinski and Smolarski (1969) determine two specific families of reverse multiples of bases not restricted to 10. Grimm and Ballew (1975) first list all base-10 reverse multiples less than $10^9$ which they have determined using a CDC 3400 (where they allow “reverse multiples” to have a zero as their first digit). Then, they present an algorithm for generating 3-digit reverse multiples of bases not restricted to 10.

10 The online bibliographic database *MathSciNet* mentions in the case of Alan Sutcliffe only one further article which has been published in the academic journal of mathematics education *The Mathematical Gazette* also in 1966; besides the three papers on reverse multiples only one from 2005 which also appeared in *Integers* in the case of Benjamin Holt; and no other work in Lev Kendrick’s case.

## References

[CR1] Ashton Z (2018) Mathematical Problem Choice and the Contact of Minds. In Zack and Schlimm (eds), Research in History and Philosophy of Mathematics. Proceedings of the Canadian Society for History and Philosophy of Mathematics/ Sociètè canadienne d’histoire et de philosophie des mathèmatiques, pp 191–203. Birkhäuser, Cham. 10.1007/978-3-319-90983-7_13

[CR2] Burg R (1916) Welche Zahlen geben in umgekehrter Ziffernfolge ein Vielfaches ihres Wertes? Sitzungsberichte der Berliner Mathematischen Gesellschaft 15:8–18

[CR3] Dutilh Novaes C (2019) The Beauty (?) of Mathematical Proofs. In: Aberdein and Inglis (eds), Advances in Experimental Philosophy of Logic and Mathematics, London: Bloomsbury Academic, pp 63–93

[CR4] Ernest P (1998) Social Constructivism as a Philosophy of Mathematics. State University of New York Press, Albany

[CR5] Experimental mathematics (2013) October 10. 2178 And All That Part 1. [Video]. YouTube. https://www.youtube.com/watch?v=LykiXBgwY4E &ab_channel=Experimentalmathematics

[CR6] Golomb SW (2014) Review of ‘2178 and all that’ by N. Sloane. Mathematical Reviews MR3214374

[CR7] Grimm CA, Ballew DW (1975) Reversible multiples. J Recreat Math 8(2):89–91

[CR8] Hacking I (2014) Why is there Philosophy of Mathematics at all? Cambridge University Press, Cambridge. 10.1017/CBO9781107279346

[CR9] Hardy GH (1920) Some Famous Problems of the Theory of Numbers and in particular Waring’s Problem. Clarendon Press, Oxford

[CR10] Hardy GH (1940) Mathematics in War-Time. Eureka 1(3):5–8

[CR11] Hardy GH (1940/2012) A Mathematician’s Apology, Canto ed. Cambridge University Press, Cambridge

[CR12] Hardy GH, Wright EM (1938) An Introduction to the Theory of Numbers. Clarendon Press, Oxford

[CR13] Heard J (2019) From Servant to Queen: A Journey through Victorian Mathematics. Cambridge University Press, Cambridge. 10.1017/9781316415726

[CR14] Holt BV (2014) Some general results and open questions on Palintiple numbers. Integers 14(A42):1–13

[CR15] Holt BV (2016) Derived palintiple families and their palinomials. Integers 16(A27):1–19

[CR16] Holt BV (2017) On permutiples having a fixed set of digits. Integers 17(A20):1–21

[CR17] Kaczynski TJ (1968) Note on a problem of Alan Sutcliffe. Math Mag 41(2):84–86. 10.1080/0025570X.1968.11975844

[CR18] Kendrick LH (2015) Young Graphs: 1089 et al. J Int Seq 18(15.9.7):1–27

[CR19] Klarreich E (2015) The Connoisseur of Number Sequences. Quanta Magazine Available at: https://www.quantamagazine.org/neil-sloane-connoisseur-of-number-sequences-20150806/

[CR20] Klosinski LF, Smolarski DC (1969) On the Reversing of Digits. Math Mag 42(4):208–210. 10.1080/0025570X.1969.11975962

[CR21] Laisant C-A, Lemoine É (eds) (1899) L’Intermédiaire des mathématiciens, vol VI. Gauthier-Villars, Paris

[CR22] Laisant C-A, Lemoine É, Maillet E, Grévy A-C (eds) (1908) L’Intermédiaire des mathématiciens, vol XV. Gauthier-Villars, Paris

[CR23] Ludington Young A (1992a) k-reverse multiples. Fibonacci Q 30(2):126–132

[CR24] Ludington Young A (1992b) Trees for k-reverse multiples. Fibonacci Q 30(2):166–174

[CR25] Pudwell L (2007) Digit reversal without apology. Math Mag 80(2):129–132. 10.1080/0025570X.2007.11953467

[CR26] Sa R, Alcock L, Inglis M, Tanswell FS (2023) Do mathematicians agree about mathematical beauty? Rev Philos Psychol. 10.1007/s13164-022-00669-3

[CR27] Sinclair N (2004) The roles of the aesthetic in mathematical inquiry. Math Think Learn 6(3):261–284. 10.1207/s15327833mtl0603_1

[CR28] Sinclair N (2006) The Aesthetic Sensibilities of Mathematicians. In: Sinclair N, Pimm D, Higginson W (eds) Mathematics and the Aesthetic New Approaches to an Ancient Affinity. Springer Science Business Media, New York, pp 87–104

[CR29] Sinclair N (2011) Aesthetic considerations in mathematics. J Humanistic Math 1(1):2–32. 10.5642/jhummath.201101.03

[CR30] Sloane NJA (2014) 2178 and all that. Fibonacci Q 52(2):99–120

[CR31] Sutcliffe A (1966) Integers that are multiplied when their digits are reversed. Math Mag 39(5):282–287. 10.1080/0025570X.1966.11975742

[CR32] The OEIS Foundation Inc. (2023) The On-Line Encyclopedia of Integer Sequences. Accessed September 2023, https://oeis.org/

[CR33] Thomas RSD (2017) Beauty is not all there is to aesthetics in mathematics. Philosophia Mathematica (III) 25(1):116–127. 10.1093/philmat/nkw019

[CR34] Tymoczko T (1993) Value Judgments in Mathematics: Can We Treat Mathematics as an Art? In White (ed), Essays in Humanistic Mathematics, 67–77. The Mathematical Association of America, MAA notes; no. 32

[CR35] Urquhart A (2015) Mathematical depth. Philosophia Mathematica (III) 23(2):233–241. 10.1093/philmat/nkv004

[CR36] Webster R, Williams G (2012/2013) On the trail of Reverse Divisors: 1089 and all that follow. Math Spect 45(3):96–102

[CR37] Weisgerber S (2023) Mathematical Progress – On Maddy and Beyond. Philosophia Mathematica (III) 31(1):1–28. 10.1093/philmat/nkac019

[CR38] Wilson DW (1997) December 15. Formula on Sequence A001232. In (The OEIS Foundation Inc. 2023). Accessed September 2023, https://oeis.org/search?q=A001232 &language=english &go=Search

